# Transcriptional regulatory network for sexual differentiation in fission yeast

**DOI:** 10.1186/gb-2007-8-10-r217

**Published:** 2007-10-10

**Authors:** Juan Mata, Anna Wilbrey, Jürg Bähler

**Affiliations:** 1 Cancer Research UK Fission Yeast Functional Genomics Group, Wellcome Trust Sanger Institute, Wellcome Trust Genome Campus, Cambridge CB10 1HH, UK; 2 Current address: Department of Biochemistry, University of Cambridge, Downing Site, Cambridge CB2 1QW, UK; 3 Current address: The Babraham Institute, B501, Babraham Research Campus, Cambridge CB22 3AT, UK

## Abstract

**Background:**

Changes in gene expression are hallmarks of cellular differentiation. Sexual differentiation in fission yeast (*Schizosaccharomyces pombe*) provides a model system for gene expression programs accompanying and driving cellular specialization. The expression of hundreds of genes is modulated in successive waves during meiosis and sporulation in *S. pombe*, and several known transcription factors are critical for these processes.

**Results:**

We used DNA microarrays to investigate meiotic gene regulation by examining transcriptomes after genetic perturbations (gene deletion and/or overexpression) of *rep1*, *mei4*, *atf21 *and *atf31*, which encode known transcription factors controlling sexual differentiation. This analysis reveals target genes at a genome-wide scale and uncovers combinatorial control by Atf21p and Atf31p. We also studied two transcription factors not previously implicated in sexual differentiation whose meiotic induction depended on Mei4p: Rsv2p induces stress-related genes during spore formation, while Rsv1p represses glucose-metabolism genes. Our data further reveal negative feedback interactions: both Rep1p and Mei4p not only activate specific gene expression waves (early and middle genes, respectively) but are also required for repression of genes induced in the previous waves (Ste11p-dependent and early genes, respectively).

**Conclusion:**

These data give insight into regulatory principles controlling the extensive gene expression program driving sexual differentiation and highlight sophisticated interactions and combinatorial control among transcription factors. Besides triggering simultaneous expression of gene waves, transcription factors also repress genes in the previous wave and induce other factors that in turn regulate a subsequent wave. These dependencies ensure an ordered and timely succession of transcriptional waves during cellular differentiation.

## Abstract 2

Microarray analysis of the transcriptome of fission yeast after genetic perturbation of 6 genes known to have a role in sexual differentiation reveals insights into the regulatory principles controlling the gene expression program driving this process.

## Background

Meiosis and the formation of specialized gametes are fundamental processes of sexual reproduction. Diploid cells of the fission yeast *Schizosaccharomyces pombe *undergo two meiotic nuclear divisions to produce four stress-resistant spores in response to environmental stimuli [1,2]. This sexual differentiation is accompanied and driven by an extensive gene expression program, during which a large proportion of all genes are either induced or repressed [3-5]. We have previously classified the genes that are up-regulated at least four-fold into four major clusters, which represent successive expression waves coinciding with the main biological events of the differentiation process: genes induced in response to environmental changes (starvation and pheromone-induced genes), early genes (pre-meiotic S phase and recombination), middle genes (meiotic divisions and early steps of spore formation), and late genes (spore maturation) [4].

Posttranscriptional control is involved in regulating mRNA levels during meiosis [6-8]. In addition, transcriptional control is of fundamental importance for sexual differentiation, and several transcription factors are essential for successful meiosis and spore formation. A subset of the genes induced in response to nutritional changes is controlled by the transcription factor Ste11p [3,9], while some early genes are under the control of the Rep1p transcriptional regulator [10-12]. The forkhead-family protein Mei4p controls the expression of several middle genes [13-15], and the basic leucine zipper (bZIP) transcription factors Atf21p and Atf31p control a subset of late genes [4]. It is not known, however, if other transcription factors are involved in this process, and how the activity of the different factors is regulated and coordinated to bring about the orderly succession of transcriptional waves.

Here, we investigate the regulation of meiotic genes by examining the transcriptome of cells deleted for or overexpressing genes encoding transcription factors whose expression is induced during sexual differentiation. Our data highlight the importance of combinatorial control in transcriptional regulation and indicate that the progression of the gene expression waves is achieved by transcriptional cascades and feedback interactions between transcription factors. We also identify two new transcriptional regulators involved in controlling late genes.

## Results and discussion

### Rep1p activates a subset of the early genes

The Rep1p transcription factor is involved in the regulation of several early genes required for premeiotic S phase and meiotic recombination [10-12]. However, the expression of some early genes is independent of Rep1p [11]. To better understand the role of Rep1p, we sought to systematically identify its target genes by using DNA microarrays to follow gene expression in *rep1Δ *mutant cells undergoing meiosis.

Because good synchrony is important to obtain gene expression profiles of high temporal resolution, we used cells carrying a temperature-sensitive mutation in the meiotic inhibitor Pat1p [16,17]. We arrested cells in G1 by removing the nitrogen source from the medium and induced meiosis by shifting the cells to the restrictive temperature. Although *pat1*-induced meiosis is not identical to wild-type meiosis in some respects [18], we have previously shown that the gene expression of early, middle, and late genes is similar in both types of experiments [4].

**Figure 1:**
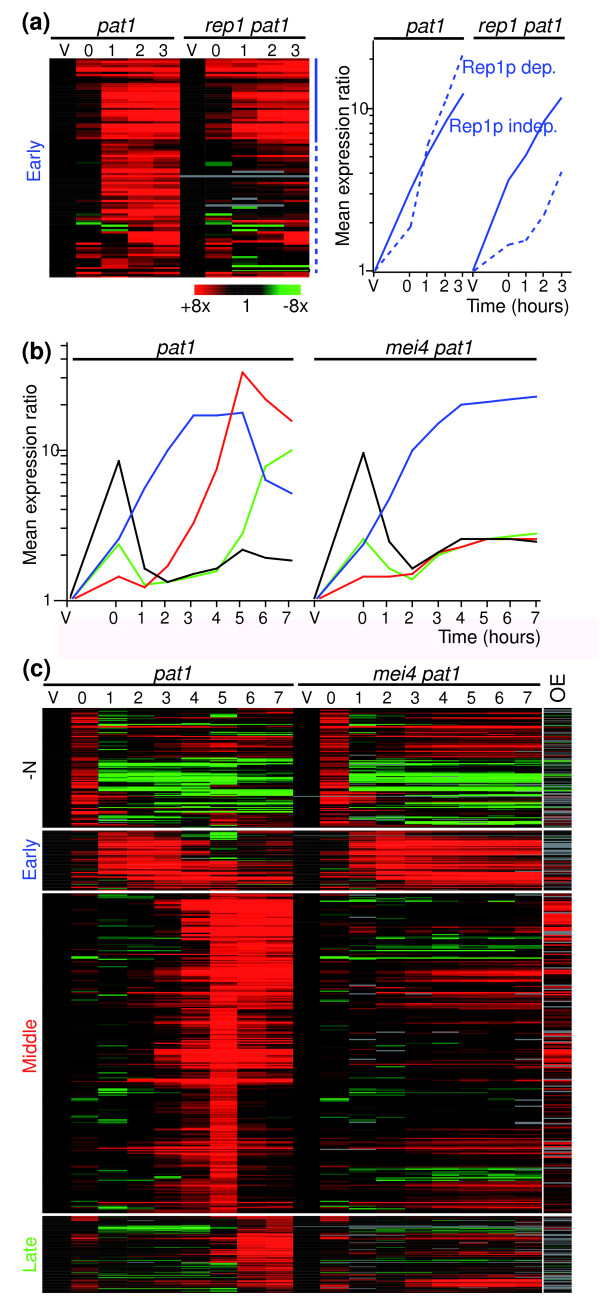
Meiotic gene expression program in *rep1Δ *and *mei4Δ *mutants. **(a) **Left: hierarchical cluster analysis with columns representing experimental time points and rows representing early genes. Vegetatively growing cells (V) are synchronized in G1 by nitrogen removal and enter meiosis by temperature shift at time 0. The mRNA levels at each time point of *pat1*(from [4]) and *rep1Δ pat1 *time courses relative to the levels in vegetative cells are color-coded as indicated at the bottom with missing data in gray. Rep1p-independent and -dependent genes are indicated at right as drawn-out or dashed blue lines, respectively. Right: average expression profiles of Rep1p-dependent and Rep1p-independent genes in wild-type (*pat1-*synchronized cells from [4]) and *rep1Δ *cells. **(b) **Average expression profiles of the four main gene clusters upregulated during *pat1*-induced meiosis: nitrogen-starvation response (black), early (blue), middle (red) and late (green), as defined in [4]. Experimental details are as in (a). The y-axis shows the expression level at the corresponding time point relative to expression in vegetative cells. Graphs from left to right: *pat1 *cells (from [4]) and *mei4Δ pat1 *cells. **(c) **Hierarchical cluster analysis with columns representing experimental time points and rows representing genes. The mRNA levels at each time point of *pat1 *time courses relative to the levels in vegetative cells are color-coded as indicated in (a). The last column (OE) shows the expression level in cells overproducing Mei4p relative to an empty vector control. The four gene clusters are indicated on the left.

Deletion of *rep1 *had complex effects on the expression of early genes (Figure 1a). About 47% of early genes were not fully induced in *rep1Δ *cells (full induction was defined as an increase within two-fold of that of wild-type cells at every time point between 1 and 3 hours; see Additional data file for complete lists of Rep1p-dependent and Rep1p-independent genes). However, the effects of *rep1Δ *on gene expression were varied, with some genes being partially induced but often in a delayed fashion compared to wild type. Rep1p-dependent and Rep1p-independent genes showed no clear functional distinction. Both groups were significantly enriched in genes involved in meiotic recombination and meiosis I (GO: 0007131 and GO: 0007127, *P *< 2 × 10^-6^), while Rep1p-dependent genes were uniquely enriched in genes involved in the mitotic cell-cycle (GO: 0000279, *P *< 7 × 10^-13^). Control of gene expression during early meiosis involves several transcription factors that also function during the mitotic cell-cycle (Cdc10p, Res2p and Rep2p), which are likely to cooperate with Rep1p [10]. The large fraction of genes whose expression seems to be independent of Rep1p suggests that additional transcription factors are important for the control of the early genes.

### Mei4p regulates the induction of middle genes

The forkhead-family transcription factor Mei4p is essential for progression through the first meiotic division and has been reported to activate the expression of some middle genes [14,15]. It is unclear, however, whether all middle genes are regulated by Mei4p or whether additional transcription factors are involved in their induction. To address this question, we used DNA microarrays to systematically identify Mei4p targets. A dual strategy was applied: first, we compared meiotic time courses of *mei4Δ *cells with wild-type cells; and second, we studied the effect of Mei4p overproduction in vegetative cells.

While deletion of *mei4 *did not impair the up-regulation of starvation-induced or early genes, the induction of almost all middle and late genes was strongly affected (Figure 1b,c). Lists of Mei4p-independent and Mei4p-dependent middle genes are presented in Additional data file 9 (Mei4p dependency was defined as an induction at least two-fold lower than that of wild-type cells at any time point between 3 and 7 hours). It is likely that the effect on late genes is indirect, as the expression of some transcription factors regulating late genes is dependent on Mei4p(see below). To distinguish between direct and indirect effects, we took advantage of the finding that overproduction of Mei4p leads to induction of its targets even in vegetative cells [14,15]. We cloned the *mei4 *gene under the control of the regulatable *nmt1 *promoter [19] and overexpressed *mei4 *in vegetative cells, in which endogenous *mei4 *mRNA is almost absent [15]. Mei4p-overproducing cells looked normal 18 hours after induction of the *nmt1 *promoter (the time point used for microarray analysis). At later time points (24 hours), they appeared smaller than wild-type cells and many cells had lysed. A total of 454 genes were induced at least two-fold following induction of *mei4 *expression (Figure 1c). This group consisted mostly of middle genes (306), as well 35 genes of the nitrogen response, 19 early genes, and 13 late genes. The remaining genes had not been classified in the original study because they did not pass the four-fold cut-off for gene expression changes relative to vegetative cells [4]. The Mei4p-induced genes included 55% of all middle genes, but only 16% of the nitrogen response genes, 19% of the early genes, and 9% of the late genes. We noticed that many non-middle genes induced by Mei4p overproduction had complex expression profiles, with several peaks of expression or unusually broad peaks (data not shown). The regulation of these genes is probably under the control of several transcription factors, with Mei4p being responsible for their expression at the time of the meiotic divisions. Of the remaining 81 genes that were induced by Mei4p overproduction but had not been classified, 36 depended on Mei4p for their full induction (as defined above for the *mei4*Δ time course; Additional data file 6). Several of the latter showed middle gene-like expression profiles. It is also possible that Mei4p overproduction leads to the artifactual induction of targets of other forkhead transcription factors (Sep1p and Fkh2p) that normally control the periodic expression of a subset of cell cycle-regulated genes [20]. We looked at whether the expression of periodic genes in cluster 1 (which is enriched in Sep1p targets and forkhead-binding sequences) [21] was increased in Mei4p-overproducing cells. The expression of 35 genes of this cluster (out of 94) was induced by Mei4p overproduction. However, 29 of these 35 had been classified as middle genes. Similarly, 9 out of 41 Sep1p-dependent periodic targets were induced by Mei4p overexpression, but 8 of them are also middle genes. Therefore, it seems unlikely that Mei4p induces the ectopic expression of Sep1p targets.

Together, the results are consistent with the view that Mei4pdirectly controls the expression of most middle genes, and that the low expression of late genes in a *mei4 *mutant is an indirect effect. Given that some middle genes (220 out of the 504 Mei4p-dependent middle genes) are not induced by Mei4p overproduction, we cannot rule out the existence of a second factor that activates the expression of middle genes. However, because the expression of almost all middle genes is reduced in a *mei4Δ *strain (504 out of 549 show at least a two-fold reduction in induction; Figure 1c), we believe this possibility to be unlikely. The strong induction of at least some middle genes, however, may be supported by posttranscriptional mechanisms [7,22].

### Control of late genes by Atf21p and Atf31p

**Figure 2:**
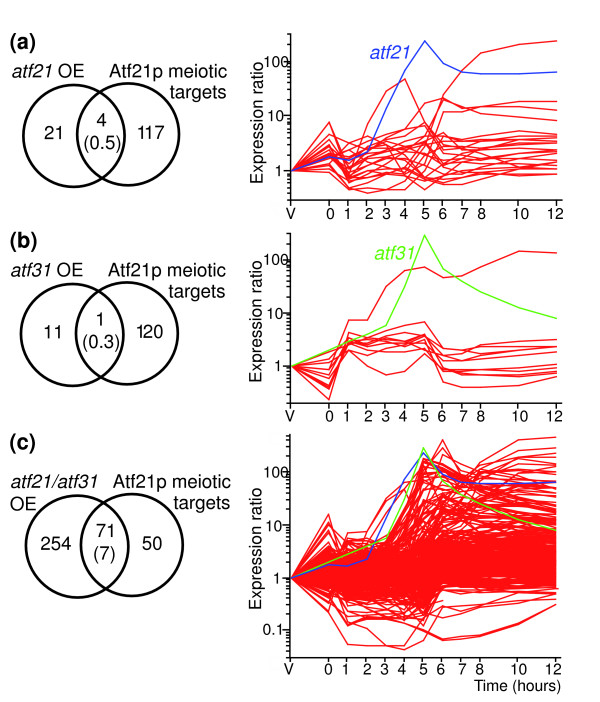
Atf21p and Atf31p cooperate to induce expression of meiotic genes. Effects of overproduction of **(a) **Atf21p, **(b) **Atf31p, and **(c) **both Atf21p and Atf31p. The Venn diagrams on the left show the overlaps between genes induced by overproduction of the different transcription factors (OE) and potential meiotic Atf21p target genes (obtained by analysis of *atf21Δ *cells [4]). The numbers in brackets show the overlap between the two lists expected by chance given the sizes of the gene sets considered and the total number of genes. The overlap between Atf31p-induced genes and meiotic Atf21p targets is not significant, while the overlaps between Atf21p and Atf21p/Atf31p-induced genes and Atf21p targets are both significant (*P *= 2.3 × 10^-3 ^and 1.5 × 10^-62^, respectively; note, however, that only four Atf21p targets are induced by Atf21p overexpression). The panels on the right show the meiotic expression profiles of genes induced by the overproduction of the corresponding transcription factors (*pat1-*synchronized cells from [4], experiment and labeling as in Figure 1). The profiles of *atf21 *and *atf31 *are highlighted in blue and green, respectively.

We have previously shown that deletion of either *atf21 *or *atf31 *affects the expression of approximately 55% of the late genes [4]. To better understand how these transcription factors function, we overexpressed them in vegetative cells, where they are normally present at low levels [4]. Overproduction of Atf21p resulted in the induction of approximately 25 genes, some of them related to stress processes [23] (Figure 2a, and a complete list is in Additional data file 7). When we took samples for microarray analysis (18 hours after induction of the *nmt1 *promoter), Atf21p-overproducing cells appeared slightly elongated. At later time points (24 hours), most cells were highly elongated and contained one or more septa (data not shown). Overproduction of Atf31p resulted in the induction of approximately 12 genes, many of which are also induced in response to heat shock [23] (Figure 2b, and a complete list is in Additional data file 7). Note that heat-shock related genes show a highly specific expression pattern during *pat1-*induced meiosis, with a sharp induction following the initial temperature shift and a quick down-regulation after the meiotic divisions [4]. Despite this finding, *atf31Δ *cells respond normally to heat shock (JM, AW and JB, unpublished observation). Atf31p-overproducing cells appeared normal after 18 hours of induction but became elongated after 24 hours (data not shown). However, neither group of genes induced by Atf21p or Atf31p overexpression showed any large overlap with the potential meiotic targets of Atf21p/Atf31p that we have previously identified [4] (Figure 2a,b, left panels).

We then simultaneously overexpressed both Atf21p and Atf31p. This caused a phenotype similar to that of cells overproducing Atf21p alone, but led to much stronger gene expression changes (325 genes induced at least two-fold), which also showed a highly significant enrichment in the meiotic targets of Atf21p/Atf31p (Figure 2c, and a complete list is in Additional data file 7). Many of the genes that were induced show expression profiles typical of late genes, but had not previously been defined as Atf21p/Atf31p targets (Figure 2c, left). There are two reasons for this difference. First, the definition of Atf21p/Atf31p targets was based on experiments using asynchronous cells, which are less sensitive. Most of the genes not originally defined as Atf21p/Atf31p targets, however, were expressed at slightly lower levels in *atf21Δ *and *atf31Δ *cells (mean expression ratios of 0.8 and 0.91, respectively) [4]. Second, the four-fold cut-off relative to vegetative cells that we used in the original study means that many of these genes were excluded from the analysis. Therefore, the real number of Atf21p/Atf31p targets is probably considerably higher than the conservative estimate from our previous study [4].

This experiment suggests that Atf21p and Atf31p cooperate to induce the expression of late genes during meiosis, probably by forming a heterodimer. These results highlight the importance of combinatorial control in the regulation of transcriptional programs. The fission yeast genome encodes three additional bZIP family transcription factors besides Atf21p and Atf31p [24-26]. These transcription factors can work as homodimers or heterodimers, creating numerous regulatory possibilities. It is likely that control by different combinations of bZIP transcription factors is used by fission yeast to launch specific gene expression programs in response to different environmental or developmental conditions. Combinatorial control by bZIP transcription factors is well known from studies in other organisms (for example, [27,28]).

### Two novel transcription factors regulate the induction of late genes

The expression of many late genes is independent of the Atf21p/Atf31p system [4]. We have previously reported that several genes that potentially encode transcription factors are specifically induced at various stages of sexual differentiation, including spore formation, raising the possibility that some of these factors regulate the expression of late genes [4].

**Figure 3:**
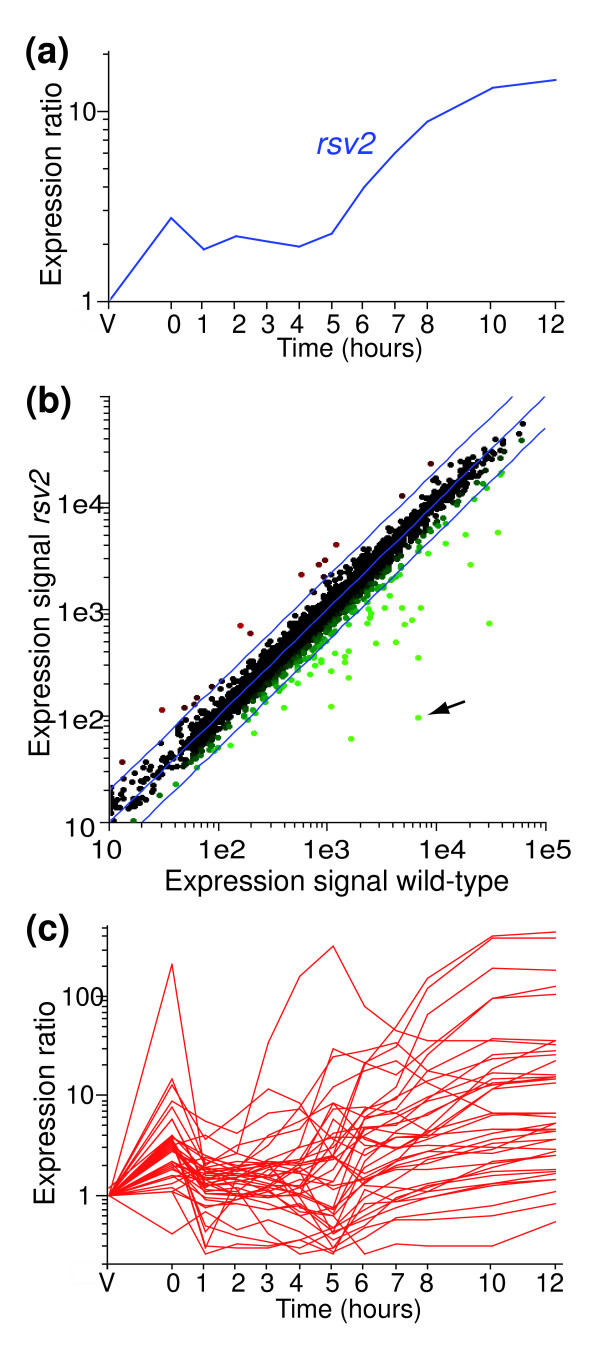
Identification of Rsv2p-dependent genes. **(a) **Expression profile of *rsv2 *during meiosis and sporulation (from [4], experiment and labeling as in Figure 1). **(b) **Comparison of gene expression levels between wild-type and *rsv2Δ *meiotic cells. Genes outside the blue lines differ by more than two-fold in expression levels. *rsv2 *is indicated by an arrow. **(c) **Expression profiles of the potential Rsv2p-dependent genes identified in (b) (*pat1-*synchronized cells from [4]).

**Figure 4:**
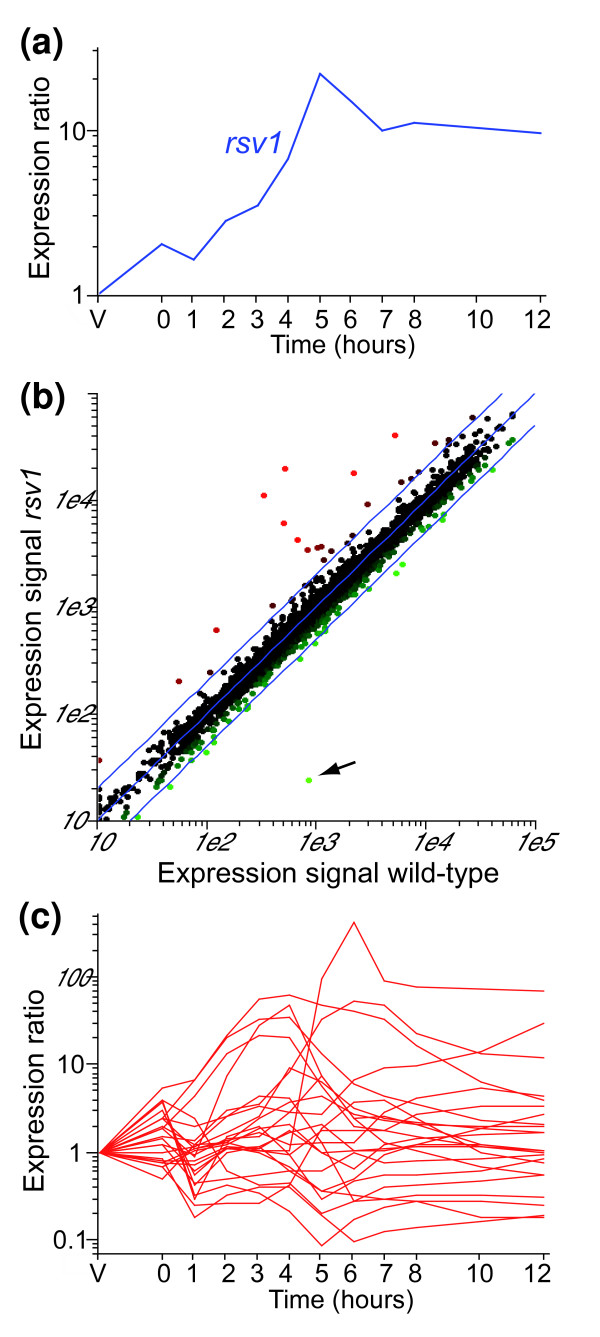
Identification of Rsv1p-dependent genes. **(a) **Expression profile of *rsv1 *during meiosis and sporulation (experiment and labeling as in Figure 1). **(b) **Comparison of gene expression levels between wild-type and *rsv1Δ *meiotic cells. Genes outside the blue lines differ by more than two-fold in expression levels. *rsv1 *is indicated by an arrow. **(c) **Expression profiles of the potential Rsv1p-dependent genes identified in (b) (*pat1-*synchronized cells from [4]).

We explored the function of *rsv1 *and *rsv2*, encoding potential transcription factors with C2H2-type zinc fingers that are induced in middle/late meiosis (Figures 3a and 4a). Rsv1p has previously been reported to be required for maintaining viability in stationary phase cells; in particular, Rsv1p is required to prevent the loss of viability associated with glucose depletion [29]. Rsv2p has a similar function during stationary phase (L López-Maury, personal communication) and during nitrogen depletion [30]. Deletion of either *rsv1 *or *rsv2 *did not affect cell viability or growth of vegetative cells, and *rsv1Δ *and *rsv2Δ *cells were able to mate and form spores of normal morphology that germinated with the same efficiency as that of wild-type spores (data not shown). Despite the lack of a clear meiotic phenotype, we reasoned that the deletions might cause subtle effects on gene expression.

We therefore used DNA microarrays to compare the transcriptome of mutant cells undergoing sexual differentiation with that of wild-type cells. For this experiment, we used homothallic haploid cells without *pat1 *mutation, which mate to form diploid cells before undergoing sexual differentiation. Because sexual differentiation is less synchronous in these wild-type cells, different meiotic stages can be studied in a single sample [4]. Despite the relative lack of synchrony, these experiments are highly reproducible: only 1.3% and 1.4% of the genes showed differences of more than 2-fold between two independent biological replicates of *rsv2 *and *rsv1 *experiments, respectively. Most genes were expressed at similar levels in wild-type and *rsv2Δ *cells, showing that meiotic progression and gene expression were not disrupted. However, 39 genes were expressed at lower levels in *rsv2Δ *mutants compared to wild-type cells (reduced at least two-fold in two independent repeats; Figure 3b, and a complete list of genes is in Additional data file 11). Of these genes, 18 had been classified as late genes, and most of the additional 21 genes also showed a late peak of expression (Figure 3c). The latter genes have not been classified as late genes, either because they did not pass the threshold of induction used for the classification, or because they had complex expression patterns containing multiple peaks [4]. A large fraction of the potential Rsv2p targets (17/39) are also induced in several different stress conditions [23]. We conclude that Rsv2p activates the expression of a subset of genes during late meiosis and may contribute to the acquisition of stress resistance by the spore. No obvious regulatory motifs were significantly enriched in the promoters of Rsv2-regulated genes.

Deletion of *rsv1 *did not cause major changes in gene expression, indicating that meiotic progression and gene expression were normal in the mutant. However, a group of 24 genes were consistently expressed at higher levels compared to wild-type cells (increased at least two-fold in two independent repeats; see Figure 4b and Additional data file 10 for a complete list of genes). This finding suggests that Rsv1p acts as a transcriptional repressor. Many of the induced genes are involved in carbohydrate metabolism, including genes for an inducer of gluconate transport (*gti1*), four hexose transporters (*ght1*, *ght3*, *ght4*, and *ght8*), a hexokinase (*hsx2*), 6-phosphogluconate dehydrogenase (*SPB660.16*), and a glucose-6-phosphate dehydrogenase (*SPCC794.01c*). The promoters of these genes were enriched in several GC-rich motifs, which might define Rsv1p-binding sites (see Table 1 in Additional data file 5). The potential Rsv1p target genes showed a variety of expression profiles during sexual differentiation in wild-type cells (Figure 4c), but seven of them were early genes, including six genes related to glucose metabolism. Rsv1p is similar to proteins of the *Saccharomyces cerevisiae *MIP1 family, which includes several transcription factors involved in glucose-repression [31].

Our data show that late genes are induced by at least two separate transcription factor systems (Rsv2p and Atf21p/Atf31p). Some late genes appear to be independent of both Atf21p/Atf31p and Rsv2p, suggesting that yet other transcription factors are required to activate their expression. However, because our data on Atf21p/Atf31p and Rsv2p targets are based on single time point experiments (which are somewhat less sensitive than time courses), it is possible that all late genes are regulated by these transcription factors and that we have failed to detect a dependency. It is also possible that some mRNAs of late genes are regulated exclusively at the posttranscriptional level.

Regulation by the Atf21p/Atf31p heterodimer appears to be specific for sexual differentiation, while Rsv1p and Rsv2p are used by the cells during both stationary phase and sexual differentiation. Indeed, Rsv1p and Rsv2p appear to regulate related sets of genes during meiosis and stationary phase (JM and JB, unpublished observations). Fission yeast cells thus use the same transcription factors to produce similar physiological responses (quiescence and resistance to environmental stress) in different situations (starvation and differentiation).

### Coordination of the meiotic transcriptional program

**Figure 5:**
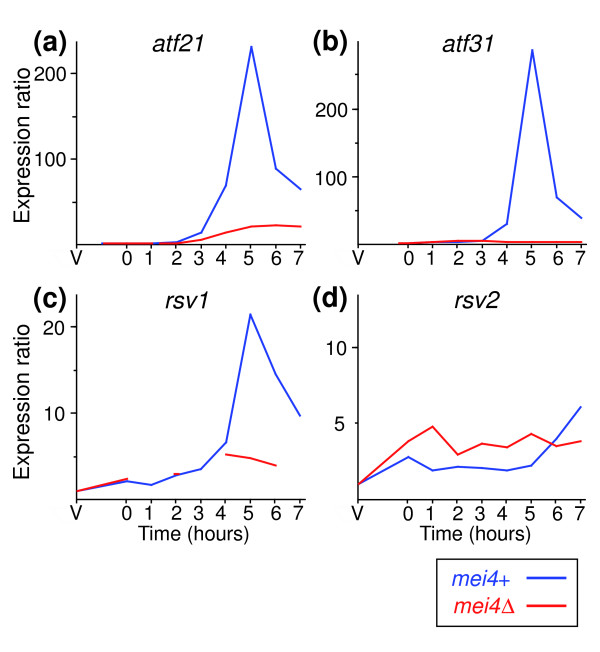
Transcription factor genes regulated by Mei4p. Expression profiles of genes encoding transcription factors in *pat1-*synchronized wild-type (blue lines, data from [4]) and *mei4Δ *(red lines) cells: **(a) ***atf21*, **(b) ***atf31*, **(c) ***rsv1 *and **(d) ***rsv2*. Experiments and labeling are as in Figure 1.

The middle genes include several putative transcriptional regulators [4], suggesting a simple model in which expression of Mei4p induces the expression of other transcription factors that, in turn, regulate the late genes. To test this hypothesis, we checked whether the expression of *atf21*, *atf31*, *rsv1 *and *rsv2 *was dependent on Mei4p. The *atf21*, *atf31* and *rsv1 *transcripts were not fully induced in the absence of Mei4p, although *atf21 *and *rsv1 *showed a small increase in *mei4Δ *cells (Figure 5a–c). This finding raises the possibility of a two-step activation system, in which only the second step is dependent on Mei4p. The expression of *rsv2*, on the other hand, is reduced in *mei4Δ *cells at late time points (7 hours; Figure 5d), and slightly induced when *mei4 *is overexpressed (2.1-fold; data not shown). Therefore, it is possible that its expression in late meiosis is at least partly under the control of Mei4p.

The alternation between transcriptional waves also requires that each wave is inactivated before the following wave is induced. We found that the reduction in the levels of early genes that normally coincides with the induction of the middle genes did not occur in *mei4Δ *mutants (Figure 1b,c). This effect has been previously observed for a small number of recombination genes [14]. We conclude that in addition to its role in up-regulating middle genes, Mei4p is necessary to switch off the early gene expression wave. This function may be indirect, as we did not observe a down-regulation of early genes when Mei4p is overproduced. The finding that the negative regulator Rsv1p seems to be a Mei4p target (Figure 5c) raises the possibility that Mei4p represses the expression of some early genes via Rsv1p. However, the inactivation of other early genes, including those involved in meiotic recombination, appears to proceed through a different, yet unknown, mechanism.

**Figure 6:**
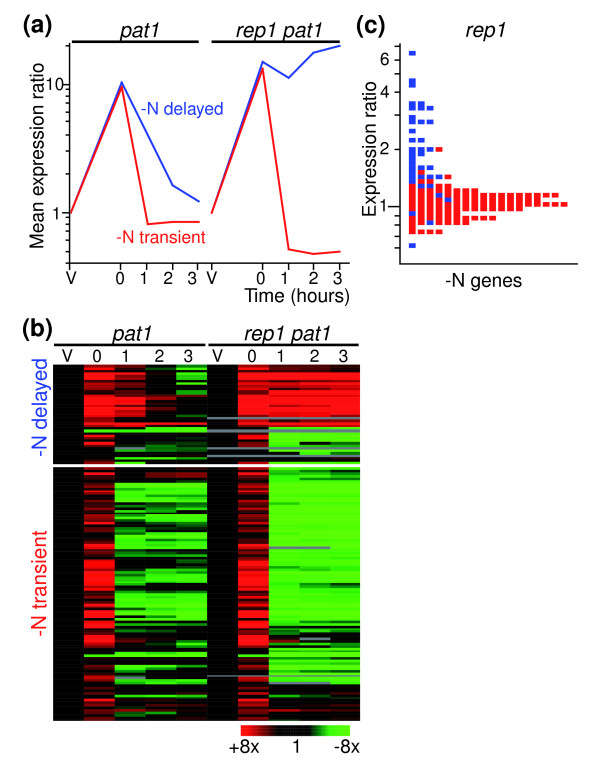
Rep1p is required to down-regulate a subset of nitrogen starvation-induced genes. **(a) **Average expression profiles in wild-type (*pat1-*synchronized cells from [4]) and *rep1Δ *cells of two subclusters of genes induced in response to nitrogen starvation: delayed (blue) and transient (red), as defined by [4]. **(b) **Hierarchical cluster analysis of the two gene clusters shown in (a), with columns representing experimental time points and rows representing genes. The mRNA levels at each time point of *pat1 *time courses relative to the levels in vegetative cells are color-coded as indicated at the bottom with missing data in gray. Labeling is as in Figure 1. **(c) **Histogram showing the gene expression levels of *rep1*Δ relative to wild-type meiotic cells. The two clusters are colored as in (a).

Similarly, we noticed that Rep1p, which is involved in the activation of a subset of early genes, is specifically required for the down-regulation of some of the genes induced in response to nitrogen starvation and pheromone signaling following entry into meiosis (Figure 6a,b). This effect is specific to the 'delayed' genes [4], which are enriched in Ste11p targets [3], while the down-regulation of other genes induced by nitrogen starvation is unaffected or even stronger in a *rep1Δ *mutant (Figure 6a,b). Although gene expression in wild-type and *pat1-*induced meiosis is similar for early, middle and late genes [4], the conditions used to trigger synchronous meiosis in *pat1 *mutants can affect the expression of genes induced by nitrogen starvation. To rule out the possibility that the effects on the expression of delayed genes were an artifact, we checked whether *rep1Δ *mutants had a similar behavior in wild-type meiosis using homothallic cells. Indeed, 'delayed' genes were expressed at higher levels in *rep1Δ *mutants compared to wild-type cells, while 'transient' genes were not affected (Figure 6c).

**Figure 7:**
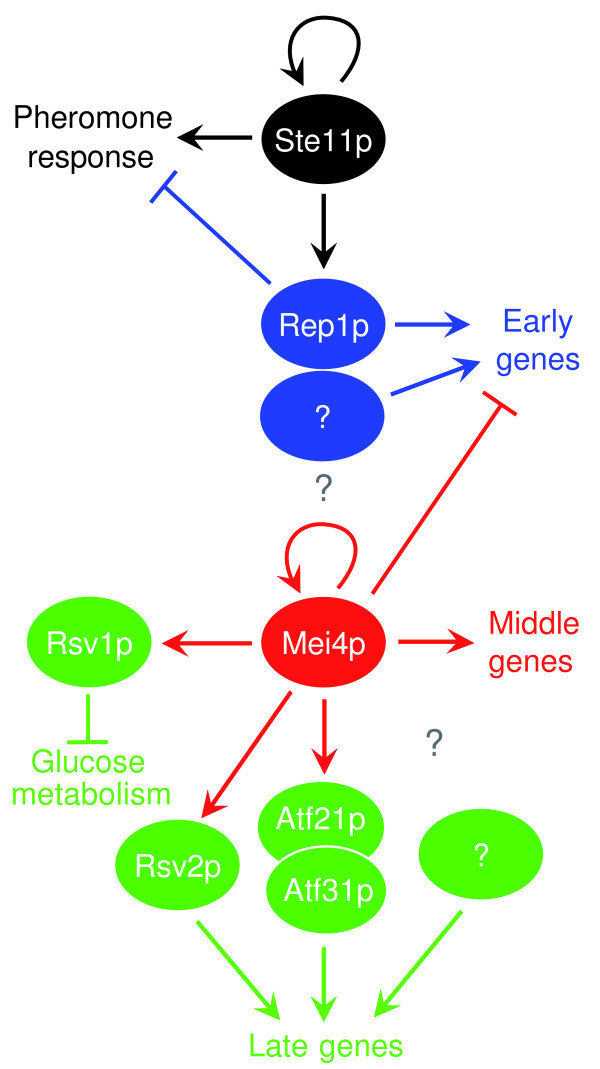
Model for transcriptional regulatory network controlling meiosis and sporulation. Arrows indicate activation and cross bars indicate repression. The colors reflect the different stages of sexual differentiation as in Figure 1. Mei4p controls transcription of its own gene in a positive feedback [14], but no other transcription factors have been identified that trigger the initial induction of *mei4*. This activation could be achieved at the posttranscriptional level [7]. Refer to the main text for further details on interactions.

These results show that the temporal pattern of successive waves of gene expression is at least partly controlled by interactions between transcription factors, in which a transcription factor induces the expression of a wave of genes, while switching off the previous wave and inducing transcription factors that will in turn trigger the next wave (Figure 7).

## Conclusion

We have analyzed the role of six transcription factors in regulating the gene expression program of different stages of sexual developmental in fission yeast. Five of these factors work as transcriptional activators, while one of them (Rsv1p) is a transcriptional repressor. In addition to a global identification of potential target genes, our results give insight into regulatory circuits that coordinate different transcriptional waves of this complex gene expression program. Two main principles are highlighted by this work (Figure 7). First, dependence between transcriptional waves is achieved by positive and negative interactions between transcription factors. Rep1p, which is transcriptionally induced by the master regulator for the pheromone response Ste11p [3,12], activates the expression of early genes but is also essential for the repression of pheromone-response genes. Similarly, Mei4p activates the expression of middle genes and is also essential for the repression of the early genes, and for the induction of genes encoding transcription factors that in turn activate the expression of late genes. These dependencies between transcription factors ensure the ordered, uni-directional succession of transcriptional waves. Second, the importance of combinatorial control: overexpression of Atf21p, Atf31p, or Atf21p/Atf31p leads to the activation of unique sets of targets. The use of different combinations of transcription factors enhances the regulatory options of cells to express specific gene sets in response to a variety of situations.

## Materials and methods

### Yeast methods and experimental design

Induction of meiosis using *pat1 *mutations was carried out exactly as in our previous study [4]. Briefly, for the *mei4Δ *deletion time course, *pat1-114/pat1-114 mei4Δ::ura4*^+^*/mei4Δ::ura4*^+^*ura4-D18/ura4-D18 ade6-M210/ade6-M216 h-/h- *diploid cells were grown in Edinburgh minimal medium containing 2% glucose (EMM) plus 0.5% NH_4_Cl, and then resuspended in EMM without NH_4_Cl (EMM-N) and incubated for 14 h at 25°C. Meiosis was started by shifting the cells to 34°C in the presence of 0.05% NH_4_Cl. The *rep1Δ *deletion time course was carried out in a similar way using *pat1-114/pat1-114 rep1Δ::ura4*^+^*/rep1Δ::ura4*^+^*ura4-D18/ura4-D18 ade6-M210/ade6-M216 h+/h+ *diploid cells. For *pat1 *time courses, RNA extracted from each time point was compared to a reference RNA prepared from *pat1-114/pat-114 *cells treated as described above to induce meiosis. The reference consisted of equal amounts of RNA extracted from vegetative cells and cells at 0, 1, 2, 3, 4, 5, 6, 7, 8, 10 and 12 hours after the temperature shift. The expression ratios at each time point were normalized to those of vegetative cells of the corresponding strain.

We deleted *rsv1 *and *rvs2 *in a homothallic *h90 *background using the one-step PCR method [32]. Meiosis in the mutants was induced by incubating cells in EMM containing 0.5% glucose without NH_4_Cl at 28°C. Samples were harvested after 15 h. Wild-type *h90 *cells treated in parallel in exactly the same way were used as a reference. For overexpression experiments, the coding sequences of the *mei4*, *atf21 *and *atf31 *genes were amplified by PCR and cloned in the pREP3X vector [33], which contains the inducible *nmt1 *promoter and a *LEU2 *selectable marker. For the co-overexpression experiment, *atf31 *was cloned into pREP4X (also under *nmt1 *control, but with an *ura4 *selectable marker). Single plasmids were transformed into a *leu1-32 h- *strain, and both plasmids were co-transformed into *leu1-32 ura4-D18 h- *cells. To induce the *nmt1 *promoter, cells were grown in EMM containing 15 μM thiamine, washed three times in EMM, and incubated at 32°C for 18 h. In every experiment, RNA extracted from cells overexpressing a particular transcription factor was compared with RNA from cells transformed with empty vectors that were treated in exactly the same way to induce the *nmt1 *promoter.

### Microarray experiments

RNA preparation, labeling, microarray production, and data processing were performed as described [34]. Microarrays were scanned with a Genepix 4000B scanner and analyzed with GenePix software (Molecular Devices, Sunnyvale, CA, USA). Hierarchical clustering, visualization, and regulatory motif searches were done with GeneSpring (Agilent, Santa Clara, CA, USA). The significance of the overlaps between gene lists was determined assuming that the overlap between random groups follows a hypergeometric distribution. All processed and normalized data are available from our website [35], and the entire raw data sets have been deposited in Array Express [36] with accession numbers E-TABM-298, E-TABM-299, E-TABM-300 and E-TABM-301. Complete normalized data sets are also available in Additional data files 1–4. Microarray experiments of *rsv1Δ *and *rsv2Δ *deletions and overexpression of Atf21p and Atf31p transcription factors were done in duplicate (independent biological repeats including a dye swap). The RNA from the Mei4p overexpression experiment was hybridized in duplicate (technical repeat, including a dye swap). The *mei4Δ *and *rep1Δ *time courses and the wild-type *rep1Δ *experiment were carried out once.

### Validation of results

We compared our data with Northern-based experiments of gene expression in *rep1Δ *and *mei4Δ *backgrounds. The microarray results were similar to published data for 8/10 genes in *rep1Δ *and 37/42 genes in *mei4Δ *(see Tables 2 and 3 in Additional data file 5).

## Abbreviations

bZIP, basic leucine zipper; EMM, Edinburgh minimal medium.

## Authors' contributions

JM and AW carried out the experiments. JM and JB conceived the study, analyzed the data and co-wrote the paper. All authors read and approved the final manuscript.

## Additional data files

The following additional data are available with the online version of this paper. Additional data file 1 includes the complete normalized dataset for *mei4Δ pat1 *and *pat1 *time courses. Additional data file 2 includes the complete normalized dataset for *rep1Δ pat1 *and *pat1 *time courses. Additional data file 3 includes the complete normalized dataset for *rsv1Δ *and *rsv2Δ *experiments. Additional data file 4 includes the complete normalized dataset for Atf21p, Atf31p, Atf21p/Atf31p and Mei4p overexpression experiments. Additional data file 5 includes additional tables. Table 1: Potential regulatory motifs in the promoters of Rsv1p-regulated genes. Table 2: Effects of *rep1Δ *on meiotic transcription: comparison with published data. Table 3: Effects of *mei4Δ *on meiotic transcription; comparison with published data. Additional data file 6 lists middle genes classified into three groups according to the effect of *mei4*Δ and Mei4p overexpression on their expression. Additional data file 7 lists genes induced by Atf21p, Atf31p and Atf21p/Atf31p overexpression and genes induced by Atf21p/Atf31p overexpression that are also reduced in *atf21Δ *meiotic cells. Additional data file 8 lists genes induced by Mei4p overexpression, classified according to their expression profiles. Additional data file 9 lists Mei4p-dependent and Mei4p-independent middle genes based on the *mei4*Δ time course experiment. Additional data file 10 includes a complete list of potential Rsv1p targets. Additional data file 11 includes a complete list of potential Rsv2p targets. Additional data file 12 lists Rep1p-dependent and Rep1p-independent early genes based on the *rep1*Δ time course experiment.

## Supplementary Material

Additional data file 1 Complete normalized dataset for *mei4Δ pat1 *and *pat1 *time courses. Click here for file

Additional data file 2 Complete normalized dataset for *rep1Δ pat1 *and *pat1 *time courses. Click here for file

Additional data file 3 Complete normalized dataset for *rsv1Δ *and *rsv2Δ *experiments. Click here for file

Additional data file 4 Complete normalized dataset for Atf21p, Atf31p, Atf21p/Atf31p and Mei4p overexpression experiments. Click here for file

Additional data file 5 Table 1: Potential regulatory motifs in the promoters of Rsv1p-regulated genes. Table 2: Effects of *rep1Δ *on meiotic transcription: comparison with published data. Table 3: Effects of *mei4Δ *on meiotic transcription; comparison with published data. Click here for file

Additional data file 6 Middle genes classified into three groups according to the effect of *mei4*Δ and Mei4p overexpression on their expression. Click here for file

Additional data file 7 Genes induced by Atf21p, Atf31p and Atf21p/Atf31p overexpression and genes induced by Atf21p/Atf31p overexpression that are also reduced in *atf21Δ *meiotic cells. Click here for file

Additional data file 8 Genes induced by Mei4p overexpression, classified according to their expression profiles. Click here for file

Additional data file 9 Mei4p-dependent and Mei4p-independent middle genes based on the *mei4*Δ time course experiment. Click here for file

Additional data file 10 Complete list of potential Rsv1p targets. Click here for file

Additional data file 11 Complete list of potential Rsv2p targets. Click here for file

Additional data file 12 Rep1p-dependent and Rep1p-independent early genes based on the *rep1*Δ time course experiment. Click here for file
