# Supplementary material for: Transcriptional regulatory network for sexual differentiation in fission yeast
Source: Genome Biol. 2007 Oct 10;8(10):R217. doi: 10.1186/gb-2007-8-10-r217 (PMC2246291; doi:10.1186/gb-2007-8-10-r217)
Supplement: Additional data file 5 — Table 1: Potential regulatory motifs in the promoters of Rsv1p-regulated genes. Table 2: Effects of rep1Δ on meiotic transcription: comparison with published data. Table 3: Effects of mei4Δ on meiotic transcription; comparison with published data. [file gb-2007-8-10-r217-S5.doc]

**Table 1**

**Potential regulatory motifs in the promoters of Rsv1p-regulated genes**

The upstream sequences of all ORFs (800 bp) were scanned for the presence of short oligomers over-represented in Rsv1p-regulated genes relative to upstream sequences of other genes, either looking for exact matches or allowing one mismatch. **Left**: Sequence of the motif. **Middle**: number of promoters that contain the motif (out of 24 potential Rsv1p targets). **Right**: probability that the observed results are due to chance.

| Exact match | | |
| --- | --- | --- |
| CCCCGC | 8 of 24 | 0.0315 |
| GCCCCGC | 5 of 24 | 0.0251 |
| 1 mismatch allowed | | |
| CCCCGCG | 12 of 24 | 0.00364 |
| GGCGGGG | 11 of 24 | 0.0278 |
| CGCCCCGC | 7 of 24 | 0.0398 |
| GCCCCGCG | 7 of 24 | 0.0113 |
| GGGCGGGG | 8 of 24 | 0.000154 |
| TCCCCCCA | 13 of 24 | 0.00197 |

**Table 2**

**Effects of *rep1∆* on meiotic transcription: Comparison with published data**

Our results are similar to published expression data for 8 out of 10 genes. In one of the remaining two cases (*rec14*), published data show a small reduction in expression, while we do not see a change. In the second case (*rec7*), we failed to detect a signal above background.

| **Biological name** | **Systematic name** | **Expression in mutant (microarray)** | **Expression in mutant (Northern)** | **Reference**  **(Northern)** |
| --- | --- | --- | --- | --- |
| *mei2* | *SPAC27D7.03c* | increased | increased | [12] |
| *res2* | *SPAC22F3.09c* | reduced | reduced | [12] |
| *cdc22* | *SPAC1F7.05* | reduced | reduced | [10,12] |
| *rec6* | *SPBC21B10.12* | reduced | reduced | [11] |
| *rec7* | *SPCC1753.03c* | not detected | reduced | [11] |
| *rec8* | *SPBC29A10.14* | reduced | reduced | [10,11] |
| *rec10* | *SPAC25G10.04c* | small reduction | reduced | [11] |
| *rec11* | *SPCC4E9.01c* | reduced | reduced | [10,11] |
| *rec14* | *SPBC32F12.02* | no change | small reduction | [11] |
| *rec15* | *SPBC1711.14* | reduced | reduced | [11] |

**Table 3**

**Effects of *mei4∆* on meiotic transcription: Comparison with published data**

There are 42 genes whose expression has been examined in a *mei4∆* background. We obtained similar results to published information for 37 genes (~90%). In one case (*meu8*) our data for wild type meiosis are not consistent with Northern experiments, while in three cases (*meu15, meu18* and *meu26*) we could not detect a reliable signal. Finally, we see a partial induction of the *meu4* gene that was not detected in the published experiment.

| **Biological name** | **Systematic name** | **Expression in mutant (microarray)** | **Expression in mutant (Northern)** | **Reference**  **(Northern)** |
| --- | --- | --- | --- | --- |
| *rec6* | *SPBC21B10.12* | Induced | Induced | [14] |
| *rec7* | *SPCC1753.03c* | Induced | Induced | [14] |
| *rec8* | *SPBC29A10.14* | Induced | Induced | [14] |
| *rec10* | *SPAC25G10.04c* | Induced | Induced | [14] |
| *dmc1* | *SPAC8E11.03c* | Induced | Induced | [14] |
| *spo5/mug12* | *SPBC29A10.02* | Induced | Induced | [14] |
| *mes1* | *SPAC5D6.08C* | Not induced | Not induced | [14] |
| *mde1* | *SPAC16E8.05C* | Not induced | Not induced | [14] |
| *mde2* | *SPBC31F10.08* | Not induced | Not induced | [14] |
| *mde3* | *SPBC8D2.19* | Not induced | Not induced | [14] |
| *mde4* | *SPBC6B1.04* | Not induced | Not induced | [14] |
| *mde5/meu30* | *SPAC25H1.09* | Not induced | Not induced | [14] |
| *mde6* | *SPAC15A10.10* | Not induced | Not induced | [14] |
| *mde7* | *SPCC320.07C* | Not induced | Not induced | [14] |
| *mde8/spn7* | *SPBC21.08C* | Not induced | Not induced | [14] |
| *mde9/spn5* | *SPAC24C9.15C* | Not induced | Not induced | [14] |
| *meu1/meu2* | *SPCC1259.14C* | Not induced | Not induced | [13] |
| *meu3* | *SPNCRNA.07* | Induced | Induced | [13] |
| *meu4/isp3* | *SPAC1F8.05* | Partial induction | Not induced | [13] |
| *meu5* | *SPAC1610.03C* | Not induced | Not induced | [13] |
| *meu6* | *SPBC428.07* | Not induced | Not induced | [13] |
| *meu7* | *SPBC16A3.13* | Not induced | Not induced | [13] |
| *meu8* | *SPCC550.10* | Not induced in wild type | Induced | [13] |
| *meu10* | *SPCC1223.12C* | Induced | Induced | [13] |
| *meu11* | *SPNCRNA.17* | Not induced | Not induced | [13] |
| *meu12/ght6* | *SPCC1235.13* | Not induced | Not induced | [13] |
| *meu14* | *SPBC1347.03* | Not induced | Not induced | [13] |
| *meu15* | *SPCPJ732.03* | Not detected | Not induced | [13] |
| *meu16* | *SPNCRNA.18* | Not induced | Not induced | [13] |
| *meu17* | *SPBC14C8.05C* | Not induced | Not induced | [13] |
| *meu18* | *SPBC409.11* | Not detected | Not induced | [13] |
| *meu19* | *SPNCRNA.29* | Partial induction | Partial induction | [13] |
| *meu20* | *SPNCRNA.30* | Partial induction | Partial induction | [13] |
| *meu21/bgs2* | *SPAC24C9.07C* | Not induced | Not induced | [13] |
| *meu22* | *SPBC19F8.06C* | Not induced | Not induced | [13] |
| *meu23* | *SPCC613.11C* | Induced | Induced | [13] |
| *meu24* | *SPCC1281.08* | Not induced | Not induced | [13] |
| *meu25* | *SPBC27.03* | Not induced | Not induced | [13] |
| *meu26* | *SPAC6B12.16* | Not detected | Not induced | [13] |
| *meu27* | *SPCC1259.14C* | Not induced | Not induced | [13] |
| *meu29* | *SPAC25H1.05* | Not induced | Not induced | [13] |
| *meu31* | *SPAC1A6.06C* | Not induced | Not induced | [13] |
